# Clinical Enhancement in AI-Based Post-processed Fast-Scan Low-Dose CBCT for Head and Neck Adaptive Radiotherapy

**DOI:** 10.3389/frai.2020.614384

**Published:** 2021-02-11

**Authors:** Wen Chen, Yimin Li, Nimu Yuan, Jinyi Qi, Brandon A. Dyer, Levent Sensoy, Stanley H. Benedict, Lu Shang, Shyam Rao, Yi Rong

**Affiliations:** ^1^ Department of Radiation Oncology, Xiangya Hospital, Central South University, Changsha, China; ^2^ Department of Radiation Oncology, University of California Davis Medical Center, Sacramento, CA, United States; ^3^ Department of Radiation Oncology, Xiamen Cancer Center, The First Affiliated Hospital of Xiamen University, Xiamen, China; ^4^ Department of Biomedical Engineering, University of California, Davis, CA, United States; ^5^ Department of Radiation Oncology, University of Washington, Seattle, WA, United States; ^6^ Department of Radiation Oncology, Mayo Clinic Arizona, Phoenix, AZ, United States

**Keywords:** deep convolutional neural network, image quality, cone beam CT, head and neck cancer, adaptive radiotherapy

## Abstract

**Purpose:** To assess image quality and uncertainty in organ-at-risk segmentation on cone beam computed tomography (CBCT) enhanced by deep-learning convolutional neural network (DCNN) for head and neck cancer.

**Methods:** An in-house DCNN was trained using forty post-operative head and neck cancer patients with their planning CT and first-fraction CBCT images. Additional fifteen patients with repeat simulation CT (rCT) and CBCT scan taken on the same day (oCBCT) were used for validation and clinical utility assessment. Enhanced CBCT (eCBCT) images were generated from the oCBCT using the in-house DCNN. Quantitative imaging quality improvement was evaluated using HU accuracy, signal-to-noise-ratio (SNR), and structural similarity index measure (SSIM). Organs-at-risk (OARs) were delineated on o/eCBCT and compared with manual structures on the same day rCT. Contour accuracy was assessed using dice similarity coefficient (DSC), Hausdorff distance (HD), and center of mass (COM) displacement. Qualitative assessment of users’ confidence in manual segmenting OARs was performed on both eCBCT and oCBCT by visual scoring.

**Results:** eCBCT organs-at-risk had significant improvement on mean pixel values, SNR (*p* < 0.05), and SSIM (*p* < 0.05) compared to oCBCT images. Mean DSC of eCBCT-to-rCT (0.83 ± 0.06) was higher than oCBCT-to-rCT (0.70 ± 0.13). Improvement was observed for mean HD of eCBCT-to-rCT (0.42 ± 0.13 cm) vs. oCBCT-to-rCT (0.72 ± 0.25 cm). Mean COM was less for eCBCT-to-rCT (0.28 ± 0.19 cm) comparing to oCBCT-to-rCT (0.44 ± 0.22 cm). Visual scores showed OAR segmentation was more accessible on eCBCT than oCBCT images.

**Conclusion:** DCNN improved fast-scan low-dose CBCT in terms of the HU accuracy, image contrast, and OAR delineation accuracy, presenting potential of eCBCT for adaptive radiotherapy.

## Introduction

Head and neck cancer (HNC) is reported as the eighth leading cause of cancer-related death worldwide (Parkin et al., 2005). HNC can have heterogeneous responses to definitive chemoradiotherapy regarding locoregional control and overall survival (Yan et al., 2012). Anatomic changes due to tumor response or weight loss may lead to under- or over-dosage to target volumes or overdosage to organs at risk (OARs) during radiotherapy. Changes in the plan dosimetry may result in increased risk of toxicity and/or impact tumor control (Chen et al., 2014; Castelli et al., 2015). In recent years, adaptive radiation therapy (ART) has been proposed to account for changes in tumor and normal organs to enhance the therapeutic ratio (Castadot et al., 2010; Schwartz, 2012). However, ART requires re-segmentation of OARs and treatment target volumes on each re-planning CT image. This process, if performed manually, is time-consuming with high intra- and inter-observer segmentation variability (Brouwer et al., 2012; Nelms et al., 2012; Lim and Leech, 2016).

Cone beam CT (CBCT) is the most common and readily available onboard imaging system for online ART (Lu et al., 2006; Woerner et al., 2017). Previous studies (Nijkamp et al., 2008; Foroudi et al., 2011) have proved that CBCT is helpful in ART for reducing the volume of irradiated healthy tissue and the dose delivered to OAR. In offline ART, CBCTs are used for anatomic change monitoring during the treatment. When needed, a new planning CT is often acquired for plan adaptation to those organ or tumor volume changes. An ideal image dataset for ART should have accurate electron density for dose calculation and high soft tissue contrast resolution for accurate and robust image registrations and/or organ segmentation. For online ART, daily images acquired for treatment alignment are used for adapting the plan to anatomic and tumor changes prior to daily treatment. Unfortunately, online adaptive CBCT is hampered by poor image quality because of scatter artifact and lack of soft-tissue contrast. Furthermore, CBCT image values have poor correlation to electron density which requires post-image processing for correction (van Zijtveld et al., 2007). Poor image quality on CBCT also limits the ability to identify organ boundaries, thus resulting in high inter-observer variability in contour delineation (Lutgendorf-Caucig et al., 2011; Altorjai et al., 2012). Deformable image registration for contours propagation has shown high uncertainties due to poor CBCT image quality (Pukala et al., 2013). Increasing scan settings might improve the image quality and electron density accuracy for CBCT images (Dyer et al., 2019), yet at a cost of increasing imaging dose to patients, which might not be trivial when adding all fractions together.

Recently, deep learning algorithms were proposed to improve CBCT image quality using different network models (Jain, 2008; Xie et al., 2012; Dong et al., 2016). Deep convolutional neural networks (DCNN) can denoise images, reduce blurring, and improve soft tissue contrast resolution (Jain, 2008; Dong et al., 2016). Specifically for those fast-scan-low-dose CBCT scans, a U-NET based DCNN was developed for enhancing image quality for HNC patients, with improved HU accuracy, signal-to-noise ratio, and small anatomical structure preservation (Yuan et al., 2019). Such image quality enhancement should bring clinical benefits specifically for ART, including improved CT-CBCT image registration accuracy, thus improved contour propagation accuracy and better visualization for identifying organs at risk on CBCT images. The present study aimed to evaluate these clinical benefits with the image quality improvements in enhanced CBCT images.

## Materials and Methods

### Patient Data

Forty post-operative HNC patients with a planning CT (pCT) and the first fraction CBCT were retrospectively identified and used for network training. A 2D U-Net shape architecture with 19-layers in 5 depths was specially optimized and trained using a total of 2080 CT and CBCT slice. The network design and architecture were described in the previous study (Yuan et al., 2019). Additional 15 patients with pCT, and replanning CT (rCT) 3–4 weeks into treatment with the same-day CBCT in relation to rCT were selected for DCNN validation. All CBCT scans were acquired with a kV x-ray imaging system mounted on a Synergy^®^ linear accelerator (Elekta AB, Stockholm, Sweden). The CT parameters were set as follows: 512 * 512 matrix size on the axial plane, 1.183 mm * 1.183 mm pixel size, and 3.0 mm thickness. CBCT parameters were set to 270 * 270 matrix size, 1.0 mm * 1.0 mm pixel size, and 3.0 mm thickness. The original CBCT (oCBCT) images were fed into the trained DCNN model to obtain enhanced image quality from CBCT images, namely eCBCT. These images are synthetic CT images created based on the CT-CBCT paired trained DCNN model.

### Organs at Risk Selection

For all patients, OARs included: left/right parotid, left/right submandibular gland (SMG), larynx, brainstem, and spinal cord. The reference contours on both pCT and rCT for each patient were manually delineated on the RayStation treatment planning system (Raysearch Laboratory, Stockholm, Sweden) by a radiation oncologist specialized in HNC and confirmed by a senior radiation oncologist. Contours on rCT were directly copied to the corresponding eCBCT and oCBCT through the gray-values based rigid image registration frame as comparison references. To eliminate the potential impact of registration differences between eCBCT and oCBCT images, the eCBCT was first registered to rCT and then the registration result of eCBCT was copied to oCBCT. All organs for delineation were completely covered in the field of CBCT view.

### Image Quality Evaluation

The manually segmented OARs on rCT was considered the ground truth for image comparison. Image quality was quantified as the difference of mean pixel values among the region of interests (ROIs) between rCT and CBCT (oCBCT, eCBCT) images, denoted ROI_m_. Seven ROIs (left/right parotid, left/right SMG, larynx, brainstem, spinal cord) were used for all patients.

The definition for signal-to-noise-ratio (SNR) is the ratio of signal power to noise power. The structural similarity index measure (SSIM) is the similarity between two images by comprehensively evaluating different properties such as luminance, contrast, and structure, which is one of human visual system-based metrics. The SNR and the SSIM of CBCTs were measured based on the seven ROIs used in the calculation of spatial non-uniformity for each patient.

$$SNR=10\cdot \log_{10}\left[\frac{\sum^{\text{​}}\sum^{\text{​}}{\left[I_{CT}\left(x,y\right)\right]}^{2}}{\sum^{\text{​}}\sum^{\text{​}}{\left[I_{CT}\left(x,y\right)-I_{eCBCT}\left(x,y\right)\right]}^{2}}\right]$$

In the formula, $I_{CT}$ represents the CT scan slice and $I_{eCBCT}$ represents the eCBCT scan slice.

$$SSIM=\frac{\left(2\mu_{eCBCT}\mu_{CT}+C_{1}\right)\left(2\delta_{eCBCT\&CT}+C_{2}\right)}{\left(\mu_{eCBCT}^{2}+\mu_{CT}^{2}+C_{1}\right)\left(\delta_{eCBCT}^{2}+\delta_{CT}^{2}+C_{2}\right)}$$

μ represents the mean value, $\delta^{2}$ represents the variance, the parameters $C_{1}={\left(k_{1}Q\right)}^{2}$ and $C_{2}={\left(k_{2}Q\right)}^{2}$ are used to stabilize the division with weak denominators, $k_{1}=0.01$ and $k_{2}=0.02$. Q is the dynamic range of the pixel-values.

### Contour Accuracy Assessment

For each patient, the CBCT pairs (oCBCT and eCBCT) and the same day rCT were imported into RayStation treatment planning system (TPS). All oCBCTs and eCBCTs were rigid registered based on skull and spine bony anatomy to the pCTs. Subsequently, a deformable image registration was performed between pCT and CBCTs, for organ contour propagation from pCTs to CBCTs image sets (both oCBCT and eCBCT) (Weistrand and Svensson, 2015). The image similarity term measured by correlation coefficient of the anatomically constrained deformation algorithm (ANACONDA) was used for CT/CBCT image comparison/registration. The whole body structure was used to define the registration region. After contour propagation, an experienced HNC radiation oncologist reviewed contours on oCBCT and eCBCT images and made contour modification if necessary. For the same patient, the type of images was not disclosed to the user at the time of contouring to avoid observer bias among different image modalities.

Accuracy of corrected propagated contours on oCBCT and eCBCT images were evaluated against the reference contours on rCTs (Whitfield et al., 2013). Quantitative assessment includes: dice similarity coefficient (DSC), Hausdorff distance (HD), and center of mass (COM) displacement. The DSC was adopted to evaluate the overlap of volumes between two contours. And it is calculated as follows:

$$\text{DSC }=\;2\times \frac{\text{Volume}1\cap^{\text{​}}\text{Volume}2}{\text{Volume}1+\text{Volume}2}$$

Volume 1 and volume 2 represent the volumes of selected reference contours. A result of 1 means a complete overlap and a result of 0 means no overlap. The HD is to measure the max distance of all the nearest points between contours, define as:

$$\text{HD}=\text{max}\left\{\begin{array}{c} \text{min d}\left(\text{a}\right)\\ \text{a}\in \text{A} \end{array},\begin{array}{c} \text{min d}\left(\text{b}\right)\\ \text{b}\in \text{B} \end{array}\right\}$$

“a” and “b” are points in contours A and B, respectively, where $\underset{\text{a}\in \text{A}}{\min}\text{d}\left(\text{a}\right)$ is the minimum distance of all points on the contour A to points on the contour B, so as the same definition used for $\underset{\text{b}\in \text{B}}{\min}\text{d}\left(\text{b}\right)$. While the center of mass displacement (COM) acts as a metric of the overall shift between two contours. It is calculated based on the following equation:

$$\text{COM }=\;\sqrt[{2}]{{\left(x1-x2\right)}^{2}+{\left(y1-y2\right)}^{2}+{\left(z1-z2\right)}^{2}}$$

*x* (1, 2), y (1, 2), z (1,2) are coordinates of the geometric centroid of the contours in comparison (Kumarasiri et al., 2014).

To further evaluate the clinical accessibility of CBCT image quality for manual segmentation, three HNC radiation oncologists visually scored OAR structures on both eCBCT and oCBCT images using a scale 1–3 according to the following criteria: 1) the outline of the structure cannot be identified; 2) the outline of the structure can be identified with moderate difficulty; 3) the image quality is close to CT simulation and the outline of the structure can be clearly identified.

### Statistical Analysis

All Statistical analyses were performed in SPSS software version 24.0 (SPSS Inc., Chicago, IL, United States) and GraphPad version 6.0. *p* < 0.05 was considered statistically significant. The Wilcoxon test was used to compare the image quality and the contouring difference between eCBCT and oCBCT.

## Results

**FIGURE 1:**
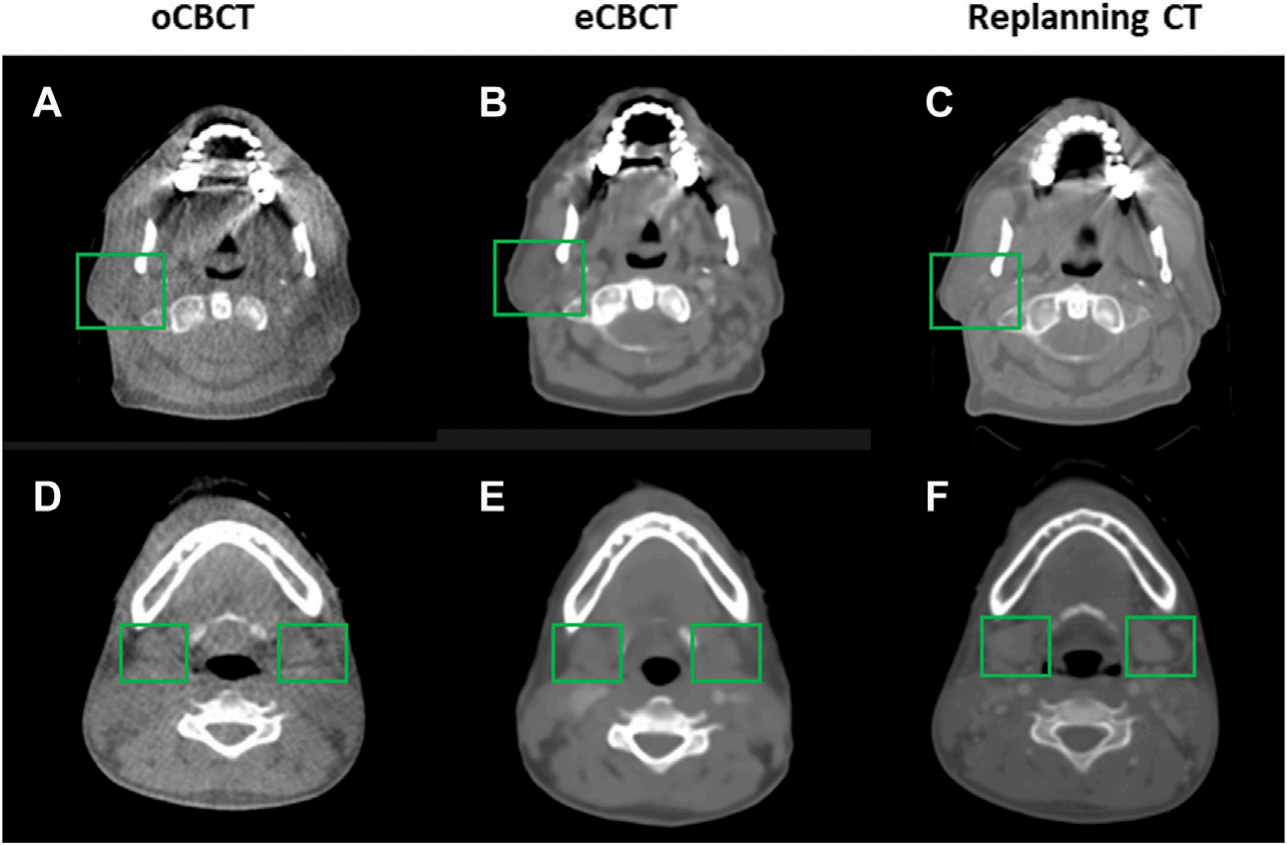
Comparison of image quality for one representative patient. eCBCT has lower image noise and less streak artifacts in the soft tissue region than the oCBCT. eCBCT also has higher image contrast than oCBCT for parotid and submandibular gland areas (see green box).

**FIGURE 2:**
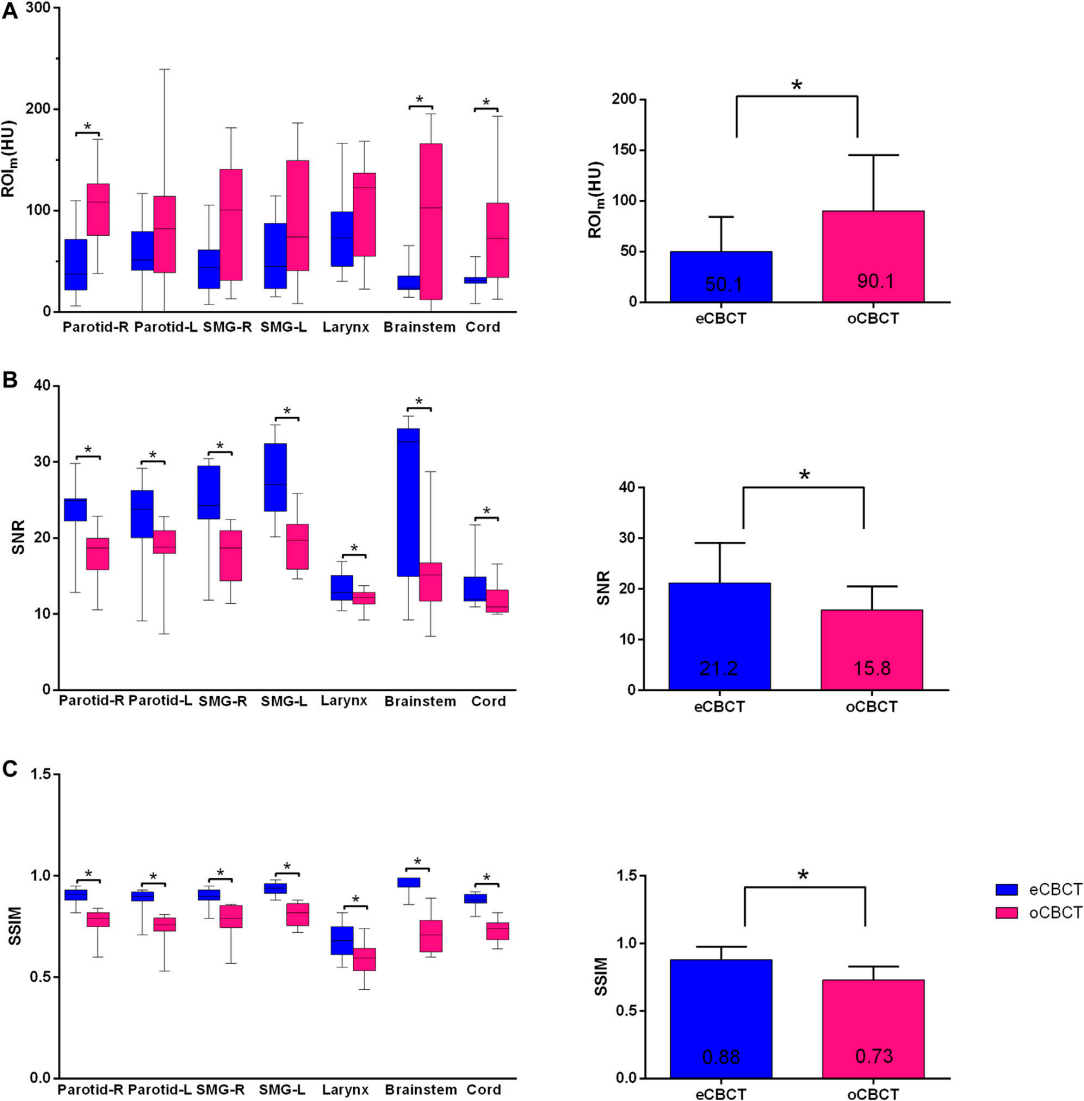
Differences in HU, SNR and SSIM between eCBCT and oCBCT. Box plots on the left side showing the ROI_m_ (HU) variations **(A)**, signal-to-noise-ratio (SNR) **(B)**, structural similarity index measure (SSIM) **(C)** for parotids, submandibular glands, larynx, brainstem, spinal cord, respectively. The limits of each box represent the 25th and 75th percentiles, the middle black line represents the median, and the upper and lower whiskers represents the highest and lowest values, respectively. The bar graphs on the right side for **(A)–(C)** showing the overall ROI_m_ (HU), SNR, SSIM variations for all organs, respectively.*Indicates that the *p* value < 0.05, and error bars are standard deviations.

Figure 1 shows image quality as an example. eCBCT images had lower noise and less streak artifacts in the soft tissue region than oCBCT. eCBCT images also had higher image contrast than oCBCT, particularly in the parotid and submandibular gland regions. A quantitative analysis of image quality for OARs is summarized in Figure 2. Seven ROIs were segmented on rCT and the mean pixel values were calculated for each ROI on rCT, oCBCT, and eCBCT images. When compared with rCT, the mean difference in CT values of ROI_m_ between rCT and oCBCT were 90 HU, while the difference between rCT and eCBCT reduced to 50 HU. This suggests that the CT values of OARs on eCBCT images more closely match those on rCT than oCBCT. When oCBCT and eCBCT SNR and SSIM were compared, eCBCT was significantly better than oCBCT (*p* < 0.05). This suggests that the DCNN method performs effectively in reducing image noise and improving image quality in eCBCT images, more closely resembling the corresponding rCT images. Metrics of image quality (ROI_m_, SNR, and SSIM) were calculated and compared for all OARs on rCT, oCBCT, and eCBCT images. We found that eCBCT showed significant improvement compared to oCBCT for all studied OARs (*p* < 0.05) (Figure 2).

**FIGURE 3:**
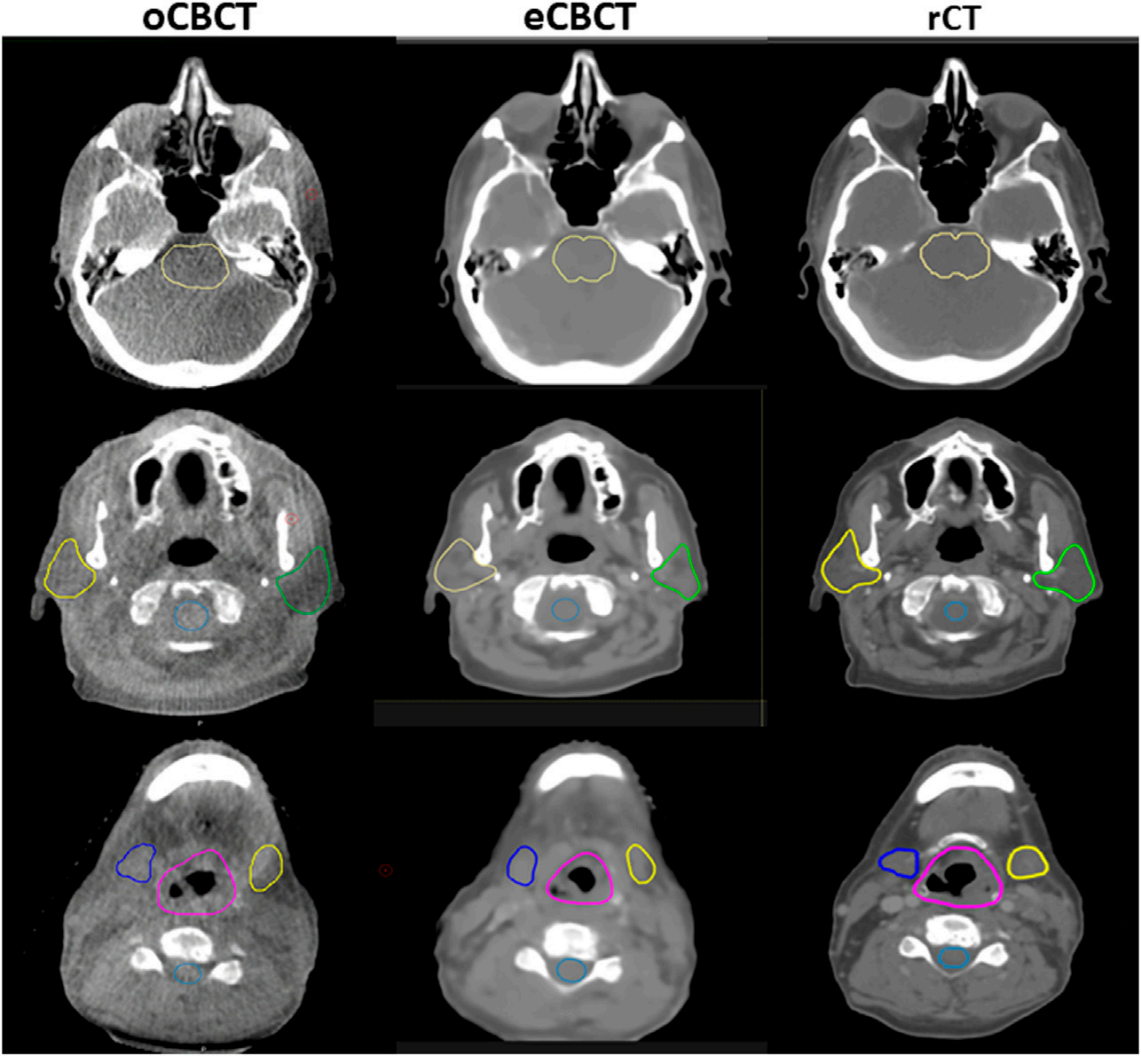
OARs delineated on transverse slices of oCBCT, eCBCT and rCT images for a representative HNC patient. OARs are outlined: brainstem (top, yellow line), parotids [middle, yellow (right) and green (left) lines], spinal cord (middle, light blue line),submandibular glands [bottom, blue (right) and yellow (left) lines], larynx (bottom, purple line).

**FIGURE 4:**
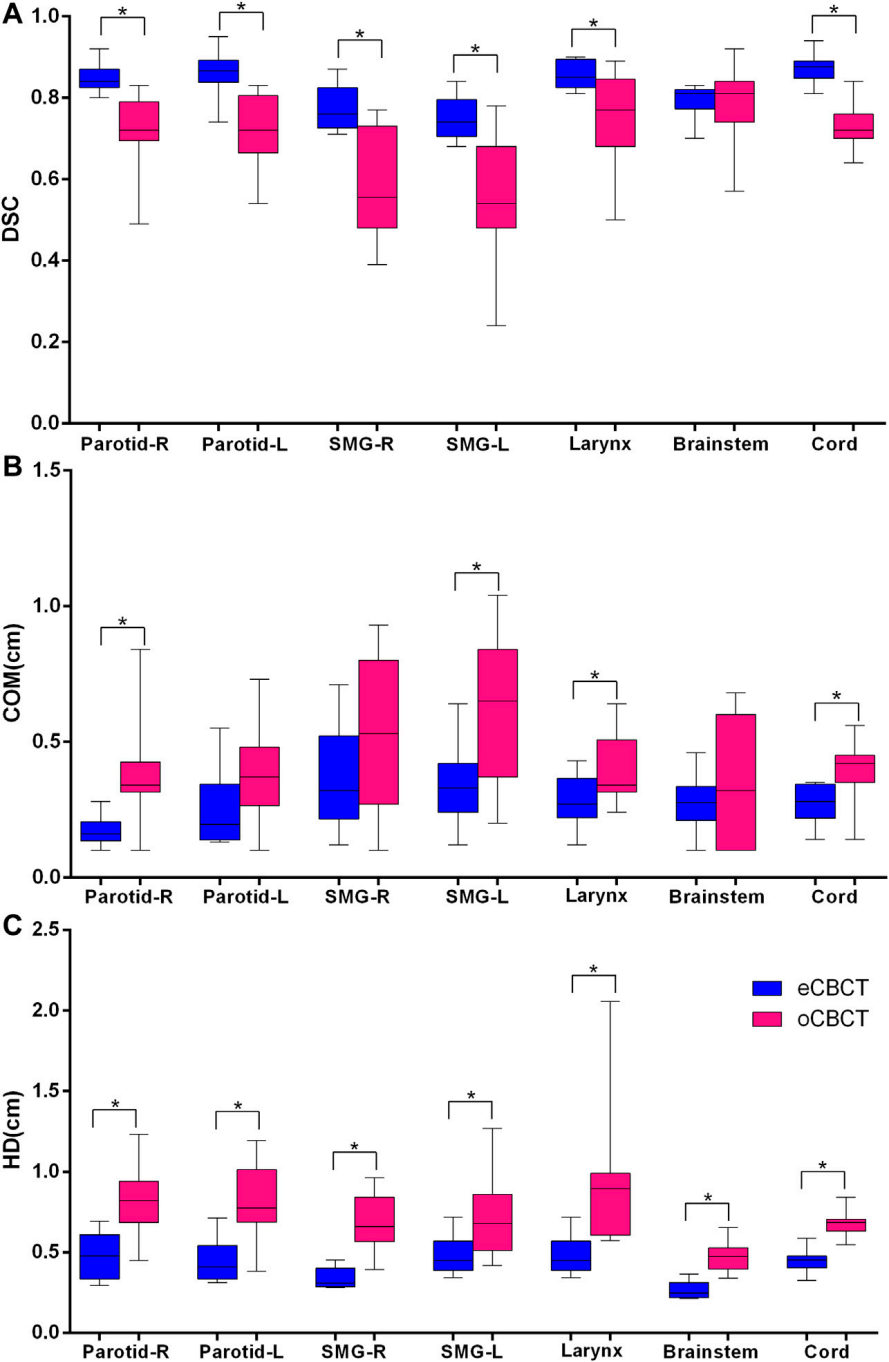
Quantitative assessment of OARs for rCTs vs. oCBCT and eCBCT images. Box plot showing Dice similarity coefficient (DSC) variations **(A)**, Center of mass (COM) displacement **(B)**, Hausdorff distance (HD) variations **(C)** for parotids, submandibular glands, larynx, brainstem, spinal cord, respectively. The limits of each box represent the 25th and 75th percentiles, the middle black line represents the median, and the upper and lower whiskers represents the highest and lowest values, respectively. *Indicates that the *p* value < 0.05.

Figure 3 shows OAR contours on transverse slices of rCT, oCBCT, and eCBCT images for one representative patient. The mean value of DSC, HD and COM difference for OARs on oCBCT and eCBCT images are shown in Figure 4. The average DSC for eCBCT-to-rCT and oCBCT-to-rCT was 0.83 ± 0.06, and 0.70 ± 0.13. The average HD for eCBCT-to-rCT was 0.42 ± 0.13 cm and for oCBCT-to-rCT was 0.72 ± 0.25 cm. The mean COM for eCBCT-to-rCT was 0.28 ± 0.19 cm and for oCBCT-to-rCT was 0.44 ± 0.22 cm eCBCT OARs had a higher DSC than oCBCT for all the structures (*p* < 0.05), except for brainstem. Similarly, the results of HD and COM all showed that OARs delineated on eCBCT were closer to rCT than oCBCT. Statistically, the difference between OARs on eCBCT vs oCBCT for HD and COM were significant for most organs. Table 1 shows the reported visual scores for OAR identification by three physicians. The scores are higher for all OAR structures on eCBCT vs oCBCT images—particularly for parotid structures. This implies that eCBCT improves ease of manual segmentation compared with oCBCT.

**TABLE 1:** Visual score (mean ± SD) for OAR segmentation ranked by three HNC physicians.

	Parotid-R	Parotid-L	SMG-R	SMG-L	Cord	Larynx	Brainstem
eCBCT	2.3 ± 0.6	2.2 ± 0.5	1.9 ± 0.3	1.9 ± 0.4	1.8 ± 0.5	1.7 ± 0.5	1.3 ± 0.5
oCBCT	1.5 ± 0.3	1.2 ± 0.4	1.1 ± 0.2	1.1 ± 0.3	1.1 ± 0.2	1.3 ± 0.4	1.1 ± 0.3

## Discussion

The studied DCNN method quantitatively improved CBCT image quality for head and neck patients. The impact of eCBCT image quality improvements in a clinical context was evaluated. SNR and SSIM of eCBCT both improved compared with those of oCBCT. An overall improvement in image quality also helped users’ judgment in identifying OARs and their subsequent contour correction on eCBCT compared with those for oCBCTs.

The inaccurate CBCT Hounsfield units will subsequently compromise dose calculation accuracy (Richter et al., 2008; Usui et al., 2013). Several approaches have been proposed to deal with the shortcomings of CBCT, such as anti-scatter grids and software-based solutions (Letourneau et al., 2007; Stankovic et al., 2017). According to Letourneau et al.’s study (Letourneau et al., 2007), they quantified the magnitude of CBCT image artifacts following the use of an anti-scatter grid and a nonlinear scatter correction. Then the corrected CBCT images were used for online planning and the dosimetric accuracy was satisfied with accepted RT standards. Veiga et al. (2014) indicated that using CT to CBCT deformable image registration provides the tools for calculating "dose of the day" without the need to obtain a new CT. However, they are limited by the time required to correct the image, and if there are large anatomical changes, these methods will also face problems due to a large challenge to the registration algorithms used in these methods. In our study, we present a fast method for intensity correction for CBCT based on a convolutional neural network. Previously, amongst those using DCNN methods, Kida et al. (2018) showed improved CBCT image quality and noise reduction for 20 prostate cancer patients using a DCNN model. Hansen et al. (2018) presented a proof-of principle of using deep learning techniques for pelvic CBCT correction and dose calculation accuracy, which is superior to conventional methods of mapping image value from the planning CT to CBCT (van Zijtveld et al., 2007), or deforming the planning CT to match a daily CBCT for the dose calculation (Veiga et al., 2015). Original CBCT often suffers from severe scatter contaminations, resulted in significant image value inaccuracy compared to that of CT. In our study, enhanced CBCT images reduced scatter artifacts, improved soft tissue contrast, and improved the HU image values within each OARs.

We compared OAR segmentation on eCBCTs and oCBCTs in reference to rCT, which was acquired on the same day as the CBCT images. Our results indicate that the eCBCTs consistently outperforms oCBCTs in all metrics. The average DSC for parotid glands in eCBCT was more than 0.80. This result is very close to previous studies. According to Zhang et al. (2014), the average DSC for parotid was 0.80 in compressed sensing based CBCT. They also proved that compressed sensing based CBCT can help to improve manual delineation of targets. Although DSC is widely used as a performance metric, it has limitation that the structure volume affects its values. Previous studies (Kumarasiri et al., 2014; Zhang et al., 2018) reported that DSC shows a positive correlation with structure volume, regardless how good the structure overlap is. Therefore, COM and HD were also used as complementary measures to better understand the quality of volume overlaps.

We chose to evaluate DCNN for CBCT image improvement in HNC patients for practical consideration. Due to the complexity of head and neck anatomic structures, and low soft tissue contrast, it is challenging to perform a manual OAR segmentation on the original CBCT. Many had attempted to create a simulated CT from deforming the planning CT to the original CBCT. However, the significant scatter artifacts on CBCT can affect the DIR accuracy. In addition, it was reported (Hou et al., 2011) that deforming contours from CT to CBCT to evaluate anatomic changes or calculate adapted dose during treatment is not reliable or requires significant manual modification. With the current CBCT image quality overall, it seems to be a common clinical practice to obtain propagated contours from the original CT to CBCT after image registration (either rigid or deformable) and correct for any obvious inaccuracy on CBCT. This of course has never been an easy task to users due to the poor quality of CBCT. Thus we included visual scoring as one of the evaluation criteria in this study. Visual score results indicated that physicians felt higher confidence in identifying the outline of those structures on eCBCT, compared to those of oCBCT.

Manual contours defined by experienced physicians were used as the comparison reference. Using manual contours as the “gold standard” is clinically feasible, and many researchers (Li et al., 2016; Zhang et al., 2014) have used this method to evaluate the delineation accuracy. A major limitation of the study is that only a small number of patients’ scans were available for this study. Future study should include more patient data and explore other anatomical regions. Moreover, contouring accuracy of gross tumor volume (GTV) on eCBCT was not studied, due to limited image quality for target delineation on both oCBCT and eCBCT. Therefore, it is worthy of noting that even though the present study has shown significant improvement toward CBCT-based ART, eCBCT image quality still has room for improvement, i.e. on the aspects of target visualization. Yet this study is still valuable for ART, in that eCBCT has improved HU accuracy and can serve for a quick on-line dose verification. The dosimetric deviation can be a trigger for ART, where a regular or high-dose CBCT can be acquired for better image quality should ART is determined necessary. This study presented that DCNN-processed low dose fast scan CBCT images, i.e. eCBCT, have the potential for head and neck adaptive radiotherapy.

## Conclusion

We validated a DCNN model for improving low-dose-fast-scan CBCT image quality, and enhanced CBCT has the potential to improve delineation accuracy for head and neck patients. These results support that enhanced CBCT has potential for adaptive radiotherapy. In addition, the CBCT image quality may still have room for improvement. Future study includes further improve the performance of the DCNN method, using enhanced CBCT for a direct dose calculation to validate the accuracy by comparing with dose distribution calculated on planning CTs.

## Data Availability Statement

The raw data supporting the conclusions of this article will be made available by the authors, without undue reservation.

## Author Contributions

YR and SR conceived and designed the study. WC and YL collected the patient’s information, WC, NY, YL, BD, JQ, SR, and LS performed the experiments. WC and NY were responsible for the data analysis. WC drafted this manuscript. YR, BD, and JQ reviewed and edited the manuscript. JQ, LS, and SB offered constructive suggestions for this study. All authors read and approved the final manuscript.
